# Nestin Null Mice Show Improved Reversal Place Learning

**DOI:** 10.1007/s11064-019-02854-w

**Published:** 2019-09-27

**Authors:** Ulrika Wilhelmsson, Marie Kalm, Marcela Pekna, Milos Pekny

**Affiliations:** 1 Laboratory of Astrocyte Biology and CNS Regeneration, Center for Brain Repair, Department of Clinical Neuroscience, Institute of Neuroscience and Physiology, Sahlgrenska Academy at the University of Gothenburg, Box 440, 40530 Gothenburg, Sweden; 2 Laboratory of Regenerative Neuroimmunology, Center for Brain Repair, Department of Clinical Neuroscience, Institute of Neuroscience and Physiology, Sahlgrenska Academy at the University of Gothenburg, 40530 Gothenburg, Sweden; 3 Florey Institute of Neuroscience and Mental Health, Parkville, VIC Australia; 4 University of Newcastle, Newcastle, NSW Australia; 5 Department of Pharmacology, Institute of Neuroscience and Physiology, Sahlgrenska Academy at the University of Gothenburg, 40530 Gothenburg, Sweden

**Keywords:** Astrocytes, Intermediate filaments, Nestin, Hippocampus, Hippocampal neurogenesis, Neural stem cells

## Abstract

The intermediate filament protein nestin is expressed by neural stem cells, but also by some astrocytes in the neurogenic niche of the hippocampus in the adult rodent brain. We recently reported that nestin-deficient (*Nes*^−/−^) mice showed increased adult hippocampal neurogenesis, reduced Notch signaling from *Nes*^−/−^ astrocytes to the neural stem cells, and impaired long-term memory. Here we assessed learning and memory of *Nes*^−/−^ mice in a home cage set up using the IntelliCage system, in which the mice learn in which cage corner a nose poke earns access to drinking water. *Nes*^−/−^ and wildtype mice showed comparable place learning assessed as the incorrect corner visit ratio and the incorrect nose poke ratio. However, during reversal place learning, a more challenging task, *Nes*^−/−^ mice, compared to wildtype mice, showed improved learning over time demonstrated by the incorrect visit ratio and improved memory extinction over time assessed as nose pokes per visit to the previous drinking corner. In addition, *Nes*^−/−^ mice showed increased explorative activity as judged by the increased total numbers of corner visits and nose pokes. We conclude that *Nes*^−/−^ mice exhibit improved reversal place learning and memory extinction, a finding which together with the previous results supports the concept of the dual role of hippocampal neurogenesis in cognitive functions.

## Introduction

The continuous generation of new neurons in the neurogenic zone in the adult hippocampus results in the reorganization of the hippocampal circuitry [1, 2], and increased hippocampal neurogenesis has been linked to better learning and increased forgetting of the acquired memories [3–5]. Astrocytes in the central nervous system (CNS) control or participate in several aspects of CNS plasticity [6–12] and some of their functions depend on astrocyte intermediate filament (known also as nanofilament) proteins GFAP, vimentin and nestin [13, 14]. The reported phenotypes resulting from the absence of the astrocyte nanofilament proteins include increased neurogenesis in the adult or aging unchallenged hippocampus [15, 16] with an effect on memory extinction [17], conceivably via the increased rate of the hippocampal circuitry reorganization, increased post-ischemic [18] and post-traumatic neurogenesis [16], improved generation and survival of neurons from neural grafts or neural stem cells after these had been grafted in the CNS of the recipient mice [19, 20], or better post-traumatic regeneration of axons and synapses [21–23]. We previously demonstrated that astrocytes from mice deficient for astrocyte intermediate filament proteins show reduced Notch signaling from astrocytes to neural stem cells [16, 24], a major mechanism that controls adult neurogenesis, and that intracellular vesicle trafficking in astrocytes depends on vimentin, GFAP, and nestin [25–28].

Nestin is expressed by neural stem cells in the adult rodent brain [29] as well as by some astrocytes in the hippocampus [28]. We recently showed that *Nes*^−/−^ mice have increased adult hippocampal neurogenesis and identified the altered Notch-signaling from *Nes*^−/−^ astrocytes to neural stem cells as the mechanism behind this phenotype [28]. As the increased hippocampal neurogenesis in *Nes*^−/−^ mice is accompanied by altered performance in the object recognition test, but no apparent effect on learning and memory as revealed by contextual and trace fear conditioning tests [28], we have here taken advantage of the continuous recordings in IntelliCage home cages to further address learning and cognitive flexibility of *Nes*^*−/−*^ mice.

## Methods

### Mice

Male mice 2–3 months old carrying a null mutation in *Nes* [28, 30] and wild-type control mice on the same genetic background (C57BL/6-129Sv) were maintained in a barrier facility with a 12 h light/dark cycle with free access of food and water. A single colony of mice was used for all the experiments, heterozygotes were used for backcrosses, and the experimental groups were generated from the next generation. Experiments were conducted according to protocols approved by the Gothenburg Ethics Committee.

### IntelliCage Housing and Set Up for Place Learning and Reversal Learning

Mice were tested for hippocampal-dependent place learning and reversal place learning using the IntelliCages (New Behavior, Zurich, Switzerland), a system for unbiased monitoring of mouse behavior in a home cage setting [31, 32]. Two weeks prior testing, mice were anesthetized with isoflurane (Abbott Laboratories, North Chicago, IL, USA) and implanted subcutaneously with micro transponders (Datamars, PetLink, Youngstown, OH, USA) to allow individual animal identification. Mice were housed in the IntelliCages in groups of 5 to 10 animals per cage. The *Nes*^−/−^ (n = 10) and wildtype (n = 13) mice had a habituation period of 4 days in the IntelliCages during which the animals were habituated to performing nose pokes to gain access to the water bottles in all the four corners of the cage. During the two first days of the habituation period, all doors were open. During the last two days of the habituation, the doors were closed and hence the mice had to practice doing a nose poke to open the door to access water. This was followed by a place learning period for which each animal was randomized to one drinking corner (the most visited corner during the habituation was excluded), in which a nose poke activated the door giving the mouse access to two water bottles. In the incorrect corners, the doors to the water bottles did not open in response to nose pokes. After 5 days, the animals were randomized to a new corner for the reversal learning and the number of visits and nose pokes for each corner were recorded. Animals were observed daily to ensure that the system registered drinking and visits. Food was provided ad libitum during the experiments in the IntelliCage and plastic houses were provided as shelters. Data from the IntelliCages were processed using the IntelliCage software (IntelliCage Plus, 2.4, New Behavior). Only the active (dark) period (16.00–08.00) was analyzed. For analysis of general activity, all visits and nose pokes during the active period were included. For analysis of place learning and reversal learning, visits not leading to a nose poke or visits lasting longer than 180 s were excluded from the analysis.

### Statistical Analysis

Values are presented as mean ± standard error of the mean (SEM). Differences were considered significant at p < 0.05. Statistical analyses of total visits and total nose pokes were performed using Mann–Whitney test in Prism 7.0 (GraphPad Software Inc., San Diego, CA, USA). Generalized estimating equations (GEE) in SPSS software (IBM, Armonk, NY, USA) were used to analyze the multiple timepoint data; this method has previously been described [32].

## Results

### ***Nes***^**−/−**^** Mice Show Increased Explorative Activity in the IntelliCage System**

**Fig. 1:**
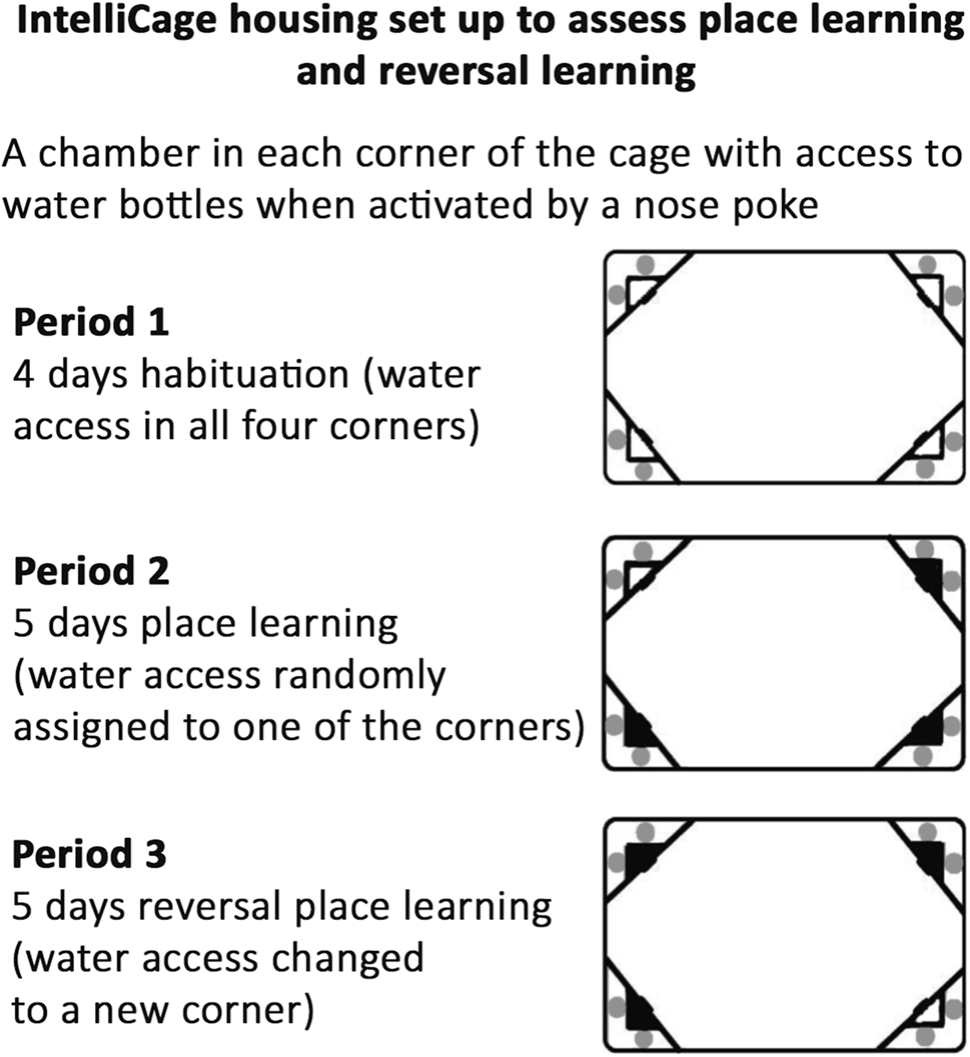
IntelliCage housing set up. Mice with micro-transponders were housed in IntelliCages and habituated for 4 days (Period 1). Next, the water access after a nose poke was randomly limited to one of the four cage corners for 5 days to assess place learning (Period 2). For assessment of reversal place learning, each mouse was assigned a new drinking corner, and the number of visits and nose pokes in each corner were recorded for 5 days (Period 3)

**Fig. 2:**
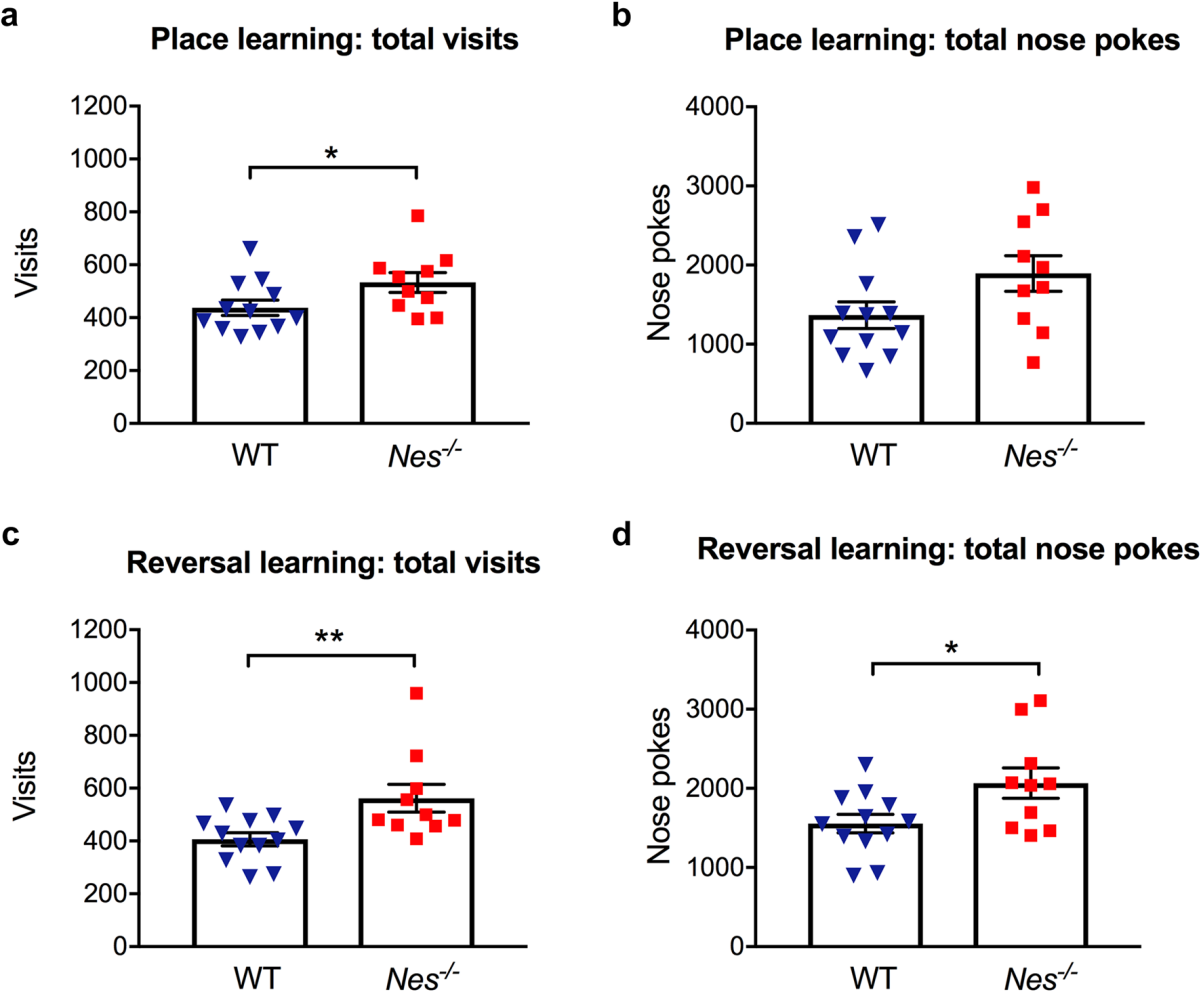
*Nes*^*−/−*^ mice show higher explorative activity in IntelliCage housing. Total number of visits to all four corners and total number of nose pokes of individual mice during place learning period (**a**, **b**) and during reversal learning period (**c**, **d**). WT, wildtype; *p < 0.05 and **p < 0.01 for *Nes*^−/−^ vs WT by Mann–Whitney test

Intellicage system allows continuous automated monitoring and data collection in a home cage setting and is used to assess place learning and the more challenging reversal place learning [17, 32]. Groups of mice were housed and monitored during several days in cages in which water is accessed by a nose poke within chambers in all four corners of the cage. During the learning trial, the water access was limited to one of the four corners. After 5 days, the water access was changed to a new corner for additional 5 days to assess reversal learning (Fig. 1). First, we assessed the general activity and behavior of the mice by quantifying all visits to the four drinking corners and all nose pokes made in the corners. Interestingly, *Nes*^−/−^ mice showed increased number of visits in the corners both during place learning and reversal learning periods compared to wildtype mice (Fig. 2a, c). The number of nose pokes was higher for *Nes*^−/−^ mice in the period of reversal learning but not in the period of place learning (Fig. 2b, d). This suggests that the overall explorative home cage activity of *Nes*^−/−^ mice was increased.

### *Nes*^−/−^ Mice Show Normal Place Learning But Better Reversal Place Learning

**Fig. 3:**
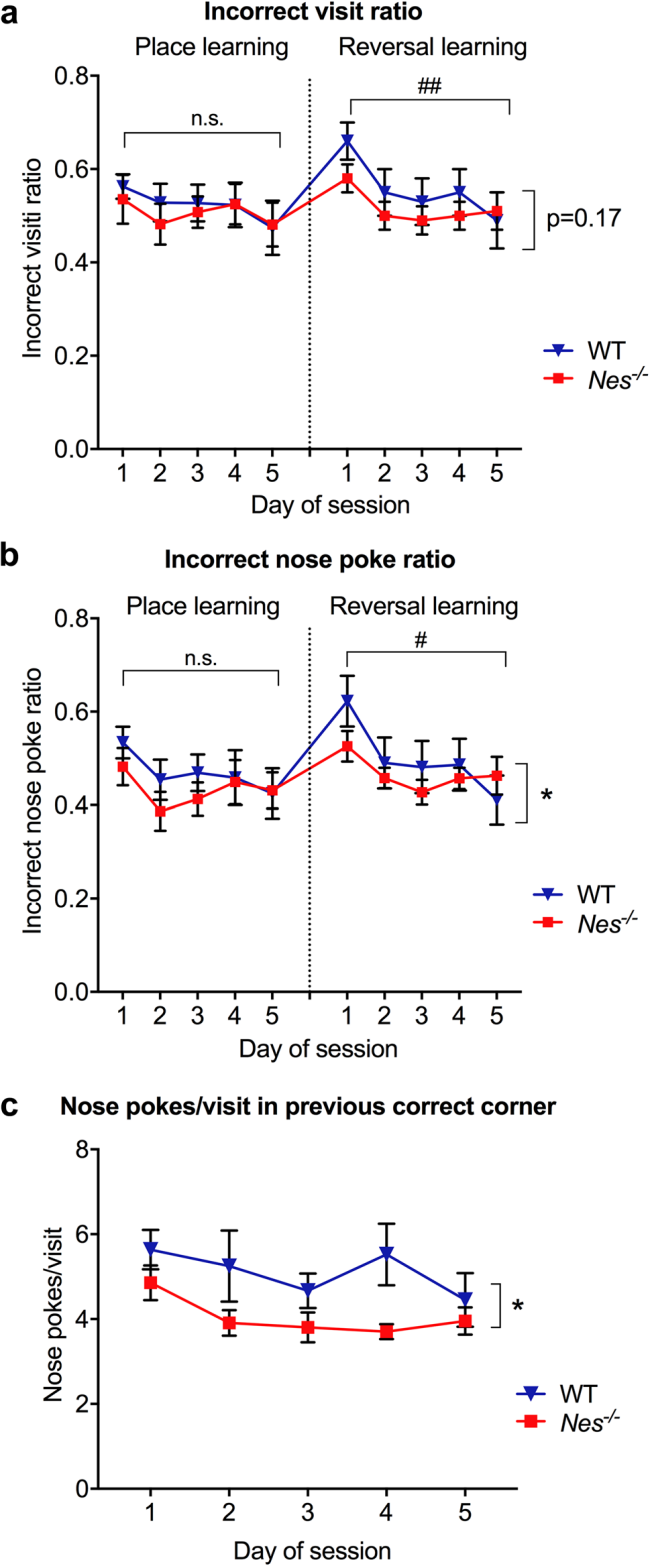
*Nes*^*−/−*^ mice show better reversal place learning in IntelliCage housing. Wildtype (WT; n = 13) and *Nes*^−/−^ mice (n = 10) showed comparable place learning as shown by the ratio of visits (**a**) and nose pokes (**b**) in the incorrect corners relative to the total number of visits and nose pokes in all four corners. When the corner with water access was changed to assess reversal learning, *Nes*^−/−^ and WT mice were comparable in terms of incorrect visit ratio (**a**), but *Nes*^−/−^ mice showed improved reversal learning in terms of incorrect nose poke ratio (**b**). During the reversal learning period, *Nes*^−/−^ mice showed better memory extinction compared to WT mice in terms of number of nose pokes per visit to the previous drinking corner (**c**). *p < 0.05 for genotype factor analyzed by generalized estimating equations. n.s., not significant; #p < 0.05 and ##p < 0.01 for time factor analyzed by generalized estimating equations

To study the ability of the mice learn and remember which of the cage corners allows access to drinking water, we quantified the visits and nose pokes to the incorrect corners relative to all the corners over time. During the place learning period, when mice had to learn that water was only available in one of the corners, *Nes*^−/−^ and wildtype mice showed comparable incorrect visit ratio (Fig. 3a) and incorrect nose poke ratio (Fig. 3b), without any improvement over time, suggesting that both groups learned and memorized the task already during the first day of place learning. To assess reversal place learning, the corner with access to water was then changed to a new corner for 5 days. *Nes*^−/−^ and wildtype mice showed decreased relative number of visits and nose pokes to the incorrect corners over time (main effect of time factor for visits p > 0.01, for nose pokes p < 0.05 by generalized estimating equations), indicating that over time the mice learned and remembered where to access water. Interestingly, in the reversal learning phase, *Nes*^−/−^ mice showed a decreased incorrect nose poke ratio over time compared to wildtype mice, while the incorrect visit ratio was comparable between the two groups of mice (Fig. 3a–b, main effect of genotype factor for nose pokes p < 0.05, for visits p = 0.17, by generalized estimating equations). To assess the memory extinction (i.e. forgetting of the previously acquired memory), we recorded the number of nose pokes per visit in the previously correct drinking corner during the reversal learning period. *Nes*^−/−^ mice showed decreased number of nose pokes per visit in the previous correct drinking corner over time compared to wildtype mice (Fig. 3c, main effect of genotype factor p < 0.05 by generalized estimating equations). These results suggest comparable place learning between *Nes*^−/−^ and wild-type mice, but improved reversal learning and memory extinction in *Nes*^−/−^ mice.

## Discussion

Here we report that *Nes*^−/−^ mice show improved learning and memory extinction in reversal place learning as assessed by continuous automated monitoring in the IntelliCage home cage system. *Nes*^−/−^ mice do not show any overt developmental CNS defects [30], but have an increased adult hippocampal neurogenesis and exhibit facilitated forgetting in the object recognition test [28]. Synaptic integration of newly formed neurons into existing hippocampal circuits was suggested to lead to improved behavioral pattern separation, i.e. the ability to distinguish between contexts or spatial cues that are similar to each other [33]. While increased number of newly formed neurons may enhance the pattern separation, the newly born neurons may also improve the cognitive flexibility and the extinction of previously learned associations, which is important in reversal learning tasks [34]. In the place learning task, *Nes*^−/−^ and wildtype mice performance was comparable, showing no improvement over time, thus suggesting that the place learning task was not challenging enough: both groups of mice learned where to find the drinking water already during the first day. Some studies have suggested that static spatial learning tasks are not dependent on the presence of adult hippocampal neurogenesis [34–36]. In contrast, the reversal place learning that depends on cognitive flexibility, showed an improvement over time for both *Nes*^−/−^ and wildtype mice, suggesting that it is a more challenging task. Neurogenesis-mediated inhibition of hippocampal neurons may enable reversal learning by reducing memory interference through the extinction of previously learned associations [37]. Intriguingly, both reversal learning assessed as incorrect nose poke ratio, and memory extinction assessed as nose pokes per visit in the previously correct corner were improved in *Nes*^−/−^ mice. These results may be a consequence of the increased survival of newly formed neurons in the hippocampus of *Nes*^−/−^ mice, although they may also depend on other changes in neuronal functioning due to the lack of nestin. The role of nestin in other aspects of CNS functions merits further investigation.

Interestingly, *Nes*^−/−^ mice showed increased explorative activity when maintained in the IntelliCage housing, as assessed by the number of visits and nose pokes in the corners of the IntelliCages. This was not detected by previous investigations that did not reveal increased locomotor activity or anxiety of *Nes*^−/−^ mice as assessed by the open field test [28] or voluntary treadmill running [38]. Thus, the automated dynamic Intellicage home cage system might be more sensitive for assessing some aspects of mouse behavior.

We conclude that the absence of nestin, previously linked to diminished Notch signaling from astrocytes to neural stem cells and increased hippocampal neurogenesis, leads to improved reversal place learning and memory extinction. These results support the scenario of the balanced trade-off between facilitated forgetting and better learning as a function of the extent of hippocampal neurogenesis that determines the dynamics of the reorganization of the hippocampal circuitry.
